# More Than Suppression: Glucocorticoid Action on Monocytes and Macrophages

**DOI:** 10.3389/fimmu.2019.02028

**Published:** 2019-08-27

**Authors:** Jan M. Ehrchen, Johannes Roth, Katarzyna Barczyk-Kahlert

**Affiliations:** ^1^Department of Dermatology, University of Münster, Münster, Germany; ^2^Institute of Immunology, University of Münster, Münster, Germany

**Keywords:** macrophage, monocyte, glucocorticoids, anti-inflammatory, resolution of inflammation

## Abstract

Uncontrolled inflammation is a leading cause of many clinically relevant diseases. Current therapeutic strategies focus mainly on immunosuppression rather than on the mechanisms of inflammatory resolution. Glucocorticoids (GCs) are still the most widely used anti-inflammatory drugs. GCs affect most immune cells but there is growing evidence for cell type specific mechanisms. Different subtypes of monocytes and macrophages play a pivotal role both in generation as well as resolution of inflammation. Activation of these cells by microbial products or endogenous danger signals results in production of pro-inflammatory mediators and initiation of an inflammatory response. GCs efficiently inhibit these processes by down-regulating pro-inflammatory mediators from macrophages and monocytes. On the other hand, GCs act on “naïve” monocytes and macrophages and induce anti-inflammatory mediators and differentiation of anti-inflammatory phenotypes. GC-induced anti-inflammatory monocytes have an increased ability to migrate toward inflammatory stimuli. They remove endo- and exogenous danger signals by an increased phagocytic capacity, produce anti-inflammatory mediators and limit T-cell activation. Thus, GCs limit amplification of inflammation by repressing pro-inflammatory macrophage activation and additionally induce anti-inflammatory monocyte and macrophage populations actively promoting resolution of inflammation. Further investigation of these mechanisms should lead to the development of novel therapeutic strategies to modulate undesirable inflammation with fewer side effects via induction of inflammatory resolution rather than non-specific immunosuppression.

## Introduction

Despite the development of many new drugs in the last decades, glucocorticoids (GCs) are still the most widely used anti-inflammatory agents in clinical medicine (1, 2). In dermatology they are indispensable to achieving rapid disease control, especially in severe autoimmune skin disorders like pemphigus vulgaris (3, 4). The use of GCs, however, is severely limited by deleterious side-effects when taken over prolonged periods of time (1, 2) Thus, in order to develop more specific anti-inflammatory strategies there is ongoing work to decipher the molecular mechanisms of GC action on immune cells.

**Figure 1:**
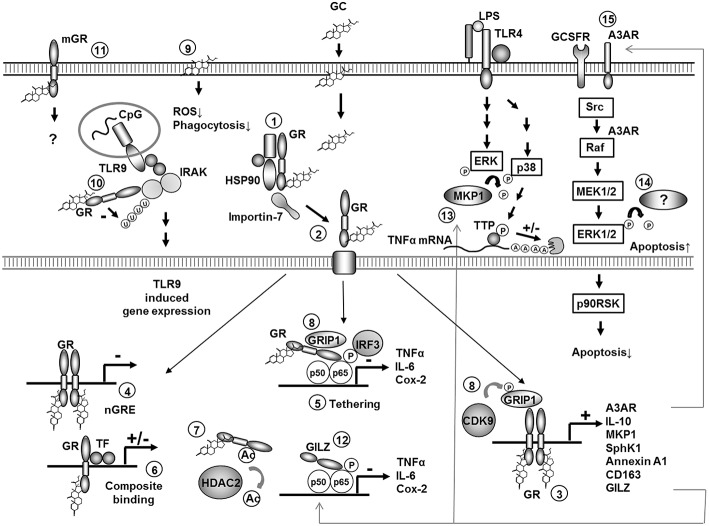
Molecular mechanisms of GC action in monocytes and macrophages. Overview of molecular mechanisms involved in regulation of gene expression by GCs in monocytes or macrophages. Specific mechanisms are discussed in the text (numbers 1–15). P, phosphorylation; Ac, acetylation; A, adenosin; U, ubiquitination; TTP, tristetraprolin; ROS, reactive oxygene species.

**Figure 2:**
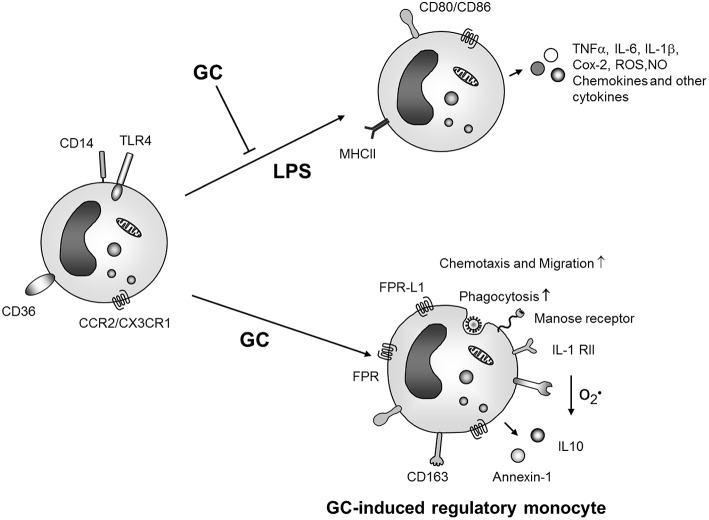
GC effects on monocyte activation and differentiation. GCs inhibit activation of monocytes by microbial products like LPS but also induce differentiation of an anti-inflammatory monocyte phenotype which produces anti-inflammatory mediators, is protected from apoptosis and shows increased migratory and phagocytic capacities. ROS, reactive oxygene species; NO, nitric oxide; IL-1 RII, IL-1 decoy receptor; FRP, formyl peptide receptor; FPR-L1, formyl peptide receptor like 1.

**Figure 3:**
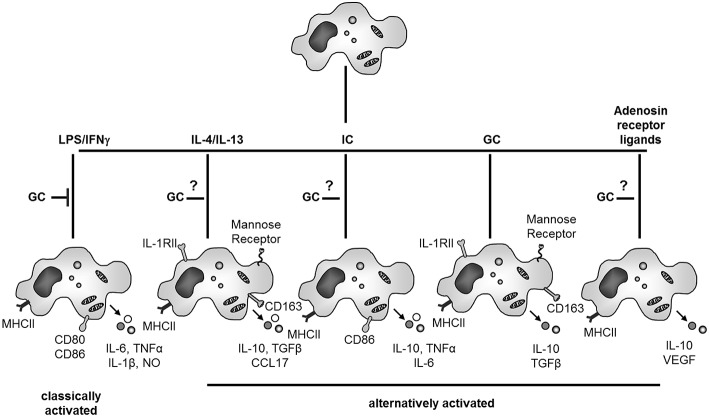
GC effects on macrophage activation and differentiation. GCs inhibit classical activation of macrophages. When acting on naïve macrophages GCs induce a specific and characteristic phenotype with anti-inflammatory properties. GC actions on other forms of alternative activation of macrophages (IL-4/IL-13, immune complexes (IC) or adenosine receptor ligands) are currently unknown. NO, nitric oxide; IL-1 RII, IL-1 decoy receptor.

GCs penetrate the plasma membrane due to their lipophilic structure and bind to the cytosolic GC-receptor (GR) which is localized in the cytoplasm in a multi-protein chaperone complex (Figure 1, 1). The GC/GR complex is then transported to the nucleus in a tightly regulated process involving conformational changes, chaperons and importins (5, 6) (Figure 1, 2). In the nucleus it can bind as a homodimer to GC response elements (GREs) in the promoter regions of GC-responsive genes. Binding to positive GREs results in increased gene transcription of the target genes, a process known as “transactivation” (Figure 1, 3). Binding to negative GREs on the other hand can result in suppression of target gene transcription (Figure 1, 4). Moreover, the monomeric GC/GR complex can inhibit gene transcription independent from DNA binding by direct interaction with other transcription factors like nuclear factor-κB (NF-κB) and activator protein-1 (AP-1)—or nuclear coactivators. These protein-protein interactions are known as tethering (Figure 1, 5). Finally, the GC/GR complex can interact with DNA (by binding to a GRE) simultaneously with another transcription factor, which is known as composite binding which may have activating or repressing effects (1, 7–9) (Figure 1, 6).

GCs act on nearly every cell of the immune system but the functional aspects of GCs differ by cell type as recently systematically confirmed in the human system (10). While GCs exhibit mainly inhibitory effects on cells of the adaptive immune system, especially on T-cells (1, 7), the effects of GCs on cells of the innate immune system are more complex. In this review we focus on GCs effects on monocytes and macrophages.

Monocytes and macrophages are a central part of the innate immune system.

They play a pivotal role in the generation of inflammatory mediators and regulation of innate and adaptive immune responses, but also contribute to resolution of inflammation, not only by producing anti-inflammatory cytokines but also by removal of pro-inflammatory pathogens, cellular debris and apoptotic cells (11–14). Thus, dysfunction of monocytes and macrophages is critically involved in the pathophysiology of severe inflammatory and autoimmune skin diseases (15–18). Specific interference with pro-inflammatory cytokines produced by monocytes and macrophages and their selective modulation by therapeutic drugs has generally revolutionized anti-inflammatory treatment strategies during the last decades (19, 20).

Blood monocytes precursors originate in the bone marrow. They enter the circulation and are present in the blood until they migrate into tissues where they can differentiate into macrophages and dendritic cells.

The development and origin of tissue macrophages has been initially thought to be dependent on these circulating monocytes. However, this long-held belief has been revised in the recent years (21). It is now widely accepted that different waves during embryonic development contribute to the tissue macrophages. The first macrophages develop from primitive macrophage progenitors in the yolk sac (YS) and give rise to microglia (22). The second wave of precursors seeding the tissues during development originate from multipotent erythro-myeloid progenitors (EMP) in the yolk sac. Most tissue macrophages are derived from these EMP. In contrast, dermal, heart, and intestinal macrophages are first seeded by embryonic liver-derived progenitors but quickly after birth they are replaced by monocytes derived from hematopoietic stem cells (23). Tissue resident macrophages show proliferative potential and self-renewal capacity and can maintain their population integrity without, or with only minimal, input from bone marrow-derived cells. The latter ones can contribute to the macrophage pool in inflammatory infiltrates and can replace tissue resident macrophages of embryonic origin e.g., after severe inflammation (24).

Tissue resident macrophages show tissue-specific functionality and adaptation to the environment. The specialization of tissue-resident macrophages and their specific gene expression profile is regulated through specific transcription factors (TFs) that finally define macrophage diversity (24, 25).

Another hallmark of macrophages is their plasticity of response phenotypes. Even during homeostasis macrophages are not in the resting state but are rather activated by microenvironmental stimuli to fulfill their function during homeostasis (26). Upon contact with pathogens macrophages express pro-inflammatory cytokines and develop an antimicrobial response. Macrophage activation is associated with profound transcriptional reprogramming (11, 27).

These data were usually generated from *in vitro* experiments using different macrophage populations. For a better comparability it is important to specify the macrophage origin. Thus, in this manuscript we refer to “murine macrophages” when cells were derived from culture of bone marrow cells in the presence of M-CSF *in vitro*. Whenever other macrophages (e.g., peritoneal macrophages) were studied, we specified this accordingly. In the human system macrophages are usually generated by *in vitro* culture of blood monocytes. When other macrophages were analyzed, this is also specified.

Depending on their stage of differentiation and on the mechanisms of activation distinct macrophage activation patterns with both pro- and anti-inflammatory functions have been described (11, 12, 28).

The classical activation of macrophages is achieved by stimulation with interferon γ (IFNγ) followed by exposure to a microbial trigger like lipopolysaccharide (LPS) and results in a pro-inflammatory phenotype. Stimulation of murine macrophages with IFNγ in the absence of a second trigger, however, results in an anti-inflammatory phenotype (29). Functionally, different types of alternatively activated macrophages are generated by exposure to triggers like IL-4 and IL-13, IL-10, or immune complexes. These macrophages, in contrast to classically activated, are particularly involved in anti-parasite responses, allergic reactions, resolution of inflammation, and tissue remodeling (11, 12, 30).

However, it was recently discovered that macrophages respond to different triggers, not only with a diverse phenotype, but can also switch from one phenotype to the other enabling them to respond to temporally and spatially dynamic activation signals (26). Thus, the above outlined dichotomous concept of macrophage polarization toward pro-inflammatory or alternative activation has been modified to a multidimensional model of macrophage activation consisting of several distinct macrophage activation programs (26, 27). Each of them is driven by specific transcriptional regulators determining a stimulus-specific gene expression pattern associated with their respective macrophage activation programs. Thus, macrophages integrate signals derived during development and signals form their environment which dictate their programing, activation, and phenotype, as well as cellular function (25–27).

Functionally and phenotypically different subpopulations or activation programs have also been demonstrated at the stage of circulating monocytes (13, 14, 31–35). In humans three different monocyte subsets can be distinguished by the differential expression of CD14 and CD16 (35). CD14^+^CD16^−^ monocytes, referred to as “classical” (or previously “inflammatory”) monocytes make up 80–90% of monocytes in human blood and during infection are rapidly recruited to sites of inflammation. The CD14^low^CD16^+^ “non-classical” monocytes are also called patrolling monocytes, are mobile in nature and contribute to the maintenance of endothelial integrity by patrolling the endothelium in search of injury. Intermediate CD14^+^CD16^+^ monocytes may develop into non-classical monocytes and had shown a high antigen presenting capacity (13). Similar subtypes have been identified in mice (31, 32, 34).

GCs are long known to act on human and murine monocytes and their successor cells (36) and there is growing evidence that they do not only influence short time mediator release but are also involved in differentiation processes resulting in an anti-inflammatory phenotype (37).

The pleiotropic effects of GCs on macrophages and monocytes including both GR-mediated repression and induction of gene transcription as well as “non-genomic” effects will be discussed in the following paragraphs.

## GC-mediated Inhibition of Pro-inflammatory Mediator Release

Inflammation is associated with the increased expression of many cytokines and other pro-inflammatory mediators. They are important for the recruitment of immune cells but can also cause severe damage in the case of uncontrolled inflammation. Monocytes and macrophages are among the most effective producers of pro-inflammatory mediators and GCs are the most powerful agents capable of limiting overwhelming and sustained inflammation. In fact, GCs inhibit the transcription of several pro-inflammatory cytokines produced by human monocytes and macrophages including IL-1β, IL-6, IL-12, TNFα, or GM-CSF and down-regulate the expression of chemokines like IL-8, RANTES, and MCP-1 (1, 38–40). GCs inhibit the synthesis of several inflammatory mediators implicated in inflammation through an inhibitory effect on enzyme production. The inducible form of nitric oxide synthase (iNOS), upregulated by pro-inflammatory cytokines, is a target of GCs in a murine macrophage cell line (J774) (41). GCs also inhibit induction of the gene coding for COX-2 in rat alveolar macrophages (42). Thus, the inhibition of pro-inflammatory mediator production from monocytes and macrophages represent a major anti-inflammatory mechanism of GCs.

The crucial importance of GC action on monocytes and macrophages for the anti-inflammatory effects of GCs *in vivo* has been elegantly demonstrated using mice with a myeloid specific depletion of the GR (GR^LysM−Cre^ mice) targeting monocytes, macrophages and also granulocytes. In these mice, the inhibitory effect of GCs on allergic contact dermatitis during the challenge phase was completely abolished while T-cell and keratinocyte specific GR depletions had no apparent effect (43). In wild-type animals GCs were very efficient in inhibiting macrophage and granulocyte infiltration during the second phase of the challenge response while leukocyte infiltration was not affected in GR^LysM−Cre^ mice. GR^LysM−Cre^ mice also demonstrated an augmentation of lethality during LPS-induced septic shock which correlated with increased expression of pro-inflammatory cytokines like TNFα, IL-6 (44), and especially IL-1β (45). These findings clearly argue for macrophages as targets for the protective role of endogenous GCs released during septic shock. Recently, it was also demonstrated that resolution of dextran sulfate sodium (DSS)-induced colitis was impaired in GR^LysM−Cre^ mice. Increased numbers of macrophages were present in colon infiltrates of GR^LysM−Cre^ and increased levels of IL-6 and decreased IL-10 concentrations were detected (46), which provides further evidence for macrophages and monocytes as important targets of the anti-inflammatory effects of GCs *in vivo*.

### Genomic Actions

The inhibitory effect of GCs on the synthesis of pro-inflammatory mediators in monocytes and macrophages both *in vitro* and *in vivo* is known for a long time and has been frequently confirmed in different species (9, 47). The direct physical inhibition of transcription factors like NF-κB and AP1 by the GC/GR complex (“transrepression”) has been proposed as the dominant mechanism for this inhibition (Figure 1, 5).

Consequently, selective GC receptor agonists (SEGRAs) which are capable of protein-protein interactions but not of DNA-binding (transactivation) have been developed (9) and have been demonstrated to be efficient in several disease models like irritant contact dermatitis in mice (48), carrageenan- and adjuvant induced arthritis (49) and experimental autoimmune neuritis in rats (50).

The importance of transrepression mechanisms *in vivo* was demonstrated in a series of elegant experiments performed in mice with a point mutation of the GC receptor which strongly impairs the ability to form receptor dimers and thus to bind to DNA (GR^dim^ mice) (47). GRE dependent induction of genes involved in gluconeogenesis is abolished in these mice while transrepression of AP-1 and NF-κB is preserved. Irritant contact dermatitis induced by phorbol esters could be suppressed by GCs in GR^dim^ mice (51). In murine peritoneal macrophages a preserved capacity of GCs to inhibit LPS-induced expression of TNFα, IL-1β, and COX-2 was observed (51). This argues for transrepression as an important mechanism for suppressing acute inflammatory responses of macrophages.

Ogawa et al. (52) analyzed the global expression pattern of genes induced by pro-inflammatory stimuli (LPS as toll like receptor (TLR) 4 ligand, as well as agonists of TLR3 and TLR9) in thioglycolate-induced murine macrophages and determined the effect of GCs on their expression. About half of the LPS induced genes were sensitive for GCs, highlighting the impressive capacity of GCs to inhibit pro-inflammatory gene expression. Disruption of p65/interferon regulatory factor (IRF)3 complexes by direct interaction of p65 with the GR seems to be the dominant mechanism for this repression (52) (Figure 1, 5). Moreover, peroxisome proliferator-activated receptor γ (PPARγ), which is one of the GC target genes in monocytes and macrophages, additionally represses overlapping but functionally distinct genes by p65/IRF3-independent mechanisms in murine ER-Hoxb8-immortalized bone marrow-derived macrophages (53). GC sensitivity was both ligand as well as gene specific, demonstrated by overlapping as well as specific inhibition patterns using other TLR agonists.

Even more complexity was added to the picture by the discovery that the GR is acetylated after GC binding and that this acetylation (Figure 1, 7) is important for GR-mediated transactivation of gene expression in murine alveolar macrophages (54). Deacetylation of the GR by histone deacetylase (HDAC) 2 on the other hand was demonstrated to be important for transrepression of p65-NF-κB activity and GM-CSF production in LPS-stimulated human alveolar macrophages. Correspondingly, a reduction in HDAC2 activity in alveolar macrophages from smokers correlated with an insensitivity to GC-mediated suppression of pro-inflammatory gene expression in these cells (55).

Moreover, transrepression of the gene transcription by GCs also involves HDAC2-mediated deacetylation of histone 4 residues, for instance, the genes coding for IL-8 or GM-CSF in a monocytic cell line (U937) or human alveolar macrophages (55, 56). Inhibition of LPS-induced NO production and iNOS expression by GCs—previously shown to involve inhibition of iNOS mRNA stability (57)—was also demonstrated to depend on HDAC activity in murine J774 macrophages (58).

Interestingly SAP30, a protein involved in stabilizing a complex which recruits HDACs to certain transcription factors was up-regulated both on the mRNA as well as on the protein level in human monocytes treated with GCs (59). This could represent a new mechanism which links GC action to deacetylation of histones or other proteins. However, inhibition of LPS-induced gene expression by GCs was independent from nuclear receptor corepressor 1 (NcoR), which represents the central part of an HDAC recruitment complex in thioglycolate-induced murine peritoneal macrophages (52). Thus, more research is needed to define the complex mechanisms by which acetylation of histones and other proteins contributes to GC action on monocytes and macrophages.

In summary, inhibition of the gene expression program induced by TLR agonists, especially by the TLR4 agonist LPS and other pro-inflammatory stimuli, is certainly an important mechanism which crucially contributes to the acute anti-inflammatory effects of GCs in many clinical settings.

There is, however, an ongoing discussion regarding the mechanisms involved in suppression of pro-inflammatory mediator production by GCs. There is accumulating evidence that transrepression of pro-inflammatory genes is only in part responsible for these effects.

In GR^dim^ mice some genes (like MKP-1) are still induced, thus a contribution of GC-induced gene transcription to the observed GC effects in the GR^dim^ mice cannot be excluded (44, 60). Moreover, in contrast to irritant contact dermatitis GCs mediated the suppression of allergic contact dermatitis is abolished in GR^dim^ mice (43). Since suppression of allergic contact dermatitis is also abolished in mice fully lacking a functional GR in myeloid cells, but not in mice with a specific deletion of the GR in T-cells, GR-dependent induction of gene-expression in macrophages seems to be important for mediating GC effects in allergic contact dermatitis (43).

The contribution of transrepression vs. other mechanisms could be gene specific as evidenced by the fact that LPS-induced expression of TNF-α, IL-6, and COX-2 but not of IL-1β, MCP-1, MIP-2, and IP-10 in macrophages was efficiently inhibited by GC treatment in GR^dim^ mice (51).

Further support for the view that the transactivation-transrepression scheme may be simplified comes from data showing that GR independent effects of some SEGRAs exist in murine macrophages (61) and data on SEGRA efficacy in clinical studies are scarce (62).

Moreover, in addition to down-regulation of pro-inflammatory mediator transcription, increased instability of the mRNAs encoding these factors can also result in reduced expression of pro-inflammatory proteins. mRNA-destabilizing factors can be induced by GR dependent gene-transcription in human monocytes (63).

In conclusion, the inhibition of pro-inflammatory mediator release depends on both transrepression, transactivation of inhibitory mechanism, and additional mechanisms like mRNA destabilization. Recently, some molecular mechanisms involved in regulating transrepression vs. transactivation have been discovered. The GR corepressor GR-interacting protein-1 (GRIP), known to be involved in GR-mediated tethering of AP1/NF-κB binding sides in macrophages, was discovered to be also crucial for GR-mediated transcription of anti-inflammatory genes. At least in murine macrophages, the last function requires phosphorylation of GRIP by CDK9 kinase while transrepression is independent from phosphorylation (64) (Figure 1, 8).

### Non-genomic Actions

Some of the effects of GCs are clearly too fast to be related to genomic actions (8). Non-genomic actions can be either receptor independent (unspecific intercalation in plasma membranes) or receptor dependent [mediated by the cytosolic GR or by a membrane bound GR (mGR)]. GCs have been described to inhibit superoxide production and phagocytosis by thioglycolate-induced and resting murine peritoneal macrophages in a receptor independent way (65, 66) (Figure 1, 9). However, there is also growing evidence for receptor dependent non-genomic GC actions, for some of which the molecular mechanism has been discovered. In murine thioglycolate-elicited peritoneal macrophages the GC/GR complex suppresses TLR9-induced cytokine production. This occurs due to the inhibition of the ubiquitination of IL1-R-associated kinase-1 (IRAK-1) by direct physical interaction between IRAK-1 and the GC/GR complex. This effect was rapid (30 min), independent from gene-transcription, and did not occur in the presence of the GR antagonist RU486. The inhibitory effect was also specific for TLR9-mediated signaling (67) (Figure 1, 10).

In human monocytes high sensitive immune-fluorescence staining has identified mGRs, which may be responsible for the rapid effects of glucocorticoids on these cells (68) (Figure 1, 11). The mGR is most likely derived from the same gene as the cytosolic GR and is up-regulated by LPS stimulation in monocytes (68) and down-regulated by GCs (69). The number of mGR positive monocytes is increased in autoimmune diseases like systemic lupus erythematodes (69) and rheumatoid arthritis where the number of mGR positive monocytes correlates with disease activity (68). However, the contribution of non-genomic effects to the overall anti-inflammatory properties of GCs *in vivo* remains to be elucidated.

## Induction of Anti-inflammatory Mediators

It becomes increasingly evident that GCs exert some of their anti-inflammatory functions in monocytes and macrophages by actively inducing synthesis of anti-inflammatory mediators (37). In naïve human monocytes genome wide expression screening revealed that GC mediated induction of gene expression including a significant number of anti-inflammatory genes was much more pronounced than suppressive effects on monocyte gene expression (59).

## Annexin A1

Annexin A1, earlier described as lipocortin-1, is the longest known anti-inflammatory mediator which is increased in monocytes and macrophages after GC treatment. Annexin A1 gene deficient mice show enhanced inflammatory responses and a reduced response to GCs *in vivo* and annexin A1 is able to mimic many aspects of GC action (70). Since annexin A1 is induced in monocytes and macrophages by GCs and also acts on these cells, annexin-A1 may be an autocrine modulator of GC action. Interestingly annexin A1 alone, similar to GCs—increases phagocytic uptake of apoptotic cells by murine and human macrophages and annexin A1-deficient murine macrophages are defective in phagocytosis (71, 72). In murine primary peritoneal macrophages GC-mediated suppression of LPS-induced IL-6 and TNFα- secretion was dependent on annexin A1 expression and mediated via expression of GC-induced leucine zipper (GILZ) (73). Annexin A1 also induces GILZ expression in macrophages from the pleural cavity *in vivo* (74) but GILZ expression is not mandatory for anti-inflammatory effects of annexin A1 during resolution of LPS-induced inflammation *in vivo* (74). The global gene expression profile of human monocytes treated with the anti-inflammatory N-terminal region of annexin A1 (75) revealed some similarities to the GC-induced expression profile in human monocytes (59) like down-regulation of CCR2 and up-regulation of amphiregulin but no major overlap, demonstrating that the exact contribution of annexin A1 induction to GC action on monocytes and macrophages still has to be determined.

## IL-10

IL-10 is an anti-inflammatory cytokine which is induced by GCs in human monocytes and murine primary peritoneal macrophages and B-cells but not in T-cells (10, 76, 77). The effects of GCs on B-cells, which are important cells in autoimmunity but not the focus of this overview, are generally poorly understood. Apart from activating effects like induction of IL-10 production also inhibitory effects e.g., on immunoglobulin production and induction of B-cell apoptosis have been reported (1).

In human monocytes and murine primary peritoneal and bone marrow macrophages the effects of GCs on IL-10 production depend on state of differentiation, the presence of a co-stimulatory factor like LPS, regulation of the GR by GCs and on GC concentration (77, 78). In addition, up-regulation of IL-10 depends on p38 MAPK activity. In murine macrophages GCs suppress p38 MAPK activity by inducing MKP-1 which is further induced by IL-10. Thus, induction of IL-10 by GCs is regulated in a very complex manner (60, 79).

IL-10 acts on human monocytes and represses pro-inflammatory cytokine production while inducing anti-inflammatory mediators including GILZ, IL-1ra, and CD163 (80, 81). A comparison of the GC and IL-10-induced genome wide expression profile in human monocytes however reveals no significant overlap and in contrast to GCs IL-10 did not increase phagocytic or migratory capacity of monocytes. Thus, GC action on human monocytes cannot be mimicked by action of IL-10 (59, 80, 82). However, IL-10 has a synergistic effect on GC-induced protection from apoptosis in human monocytes (82). IL-10 and GCs induce clearly distinct gene expression profiles in human macrophages as well (30).

## CD163

CD163 is a scavenger receptor expressed exclusively on the monocytes and macrophages-lineage and is considered a marker for alternatively activated macrophages. CD163 expression can be up-regulated by GCs, IL-10, and IL-6 in human monocytes and macrophages (83–85). The treatment of humans with a single dose of GCs resulted in fast and sustained up-regulation of CD163 expression on peripheral blood monocytes *in vivo* (86). CD163 is cleaved from the plasma membrane and is found in high amounts in human serum in several inflammatory conditions (87) including systemic sclerosis (88). CD163 is a high affinity receptor for hemoglobin-haptoglobin (Hb-Hp) complexes (89). The clearance of pro-inflammatory Hp-Hb complexes by CD163 not only contributes to the recycling of iron but is also important for resolution of inflammation. Engagement of the Hb-Hp complexes with CD163 on human macrophages induces the secretion of IL-10, which then itself can induce the expression of CD163 (90). The heme metabolites carbon monoxide (CO) and biliverdin/bilirubin have a cytoprotective activity and exhibit direct anti-inflammatory activity, respectively (91). Thus, clearance of Hb-Hp represents a very important anti-inflammatory mechanism exhibited via CD163 (89, 92). Recently a binding of damage associated molecular pattern (DAMP) proteins like HMGB-1 to Hp, and the subsequent clearance of this complex by CD163 receptor on human macrophages and degradation by heme oxygenase-1 (HO-1) has been reported. In turn, the activation of HO-1 leads to secretion of IL-10 from human macrophages thereby inducing anti-inflammatory pathways (93). Moreover, CD163 was shown to be involved in the adherence of human monocytes to endothelium, as well as in inhibition of lymphocyte proliferation *in vitro* (94, 95). Soluble CD163 inversely correlated with the number of activated T-lymphocytes in inflammatory exudates of patients suffering from rheumathoid arthritis (96). Thus, besides a role in resolution of inflammation CD163 may also affect initial steps of an adaptive immune response.

Targeting GCs to CD163 expressing macrophages represents a novel treatment strategy to circumvent GC mediated side effects and successfully attenuated fructose-induced liver inflammation in mice (97).

## Induction of Mediators Inhibiting Pro-inflammatory Signal Transduction Pathways

Up-regulation of the NF-κB inhibitor IκBα has been described as the first mechanism involving active GC-dependent gene transcription to inhibit NF-κB activity in a human monocytic cell-line (THP-1) (98). However, other mechanisms, which are more important for GC-mediated NF-κB inhibition, have been reported in the meantime. GCs up-regulate GILZ expression in human monocytes and macrophages and in thioglycolate-elicited murine peritoneal macrophages (81). GILZ is a leucine zipper containing protein which lacks DNA binding activity but is able to bind to NF-κB and suppresses NF-κB-mediated activation of gene transcription in human macrophages (81, 99). Thus, this pathway comprises an alternative mechanism for GC mediated NF-κB inhibition which depends on active gene transcription and is independent from direct GR/NF-κB interactions (Figure 1, 12).

Mitogen-activated protein (MAP)-kinase cascades (Extracellular-signal Regulated Kinases (ERK)1/2, c-Jun N-terminal kinases (JNK) and p38 MAP-kinase pathways) are critically involved in signal transduction during activation of human monocytes by microbial stimuli like LPS (100). MAP kinase phosphatase 1 (MKP-1) [also known as Dual specificity protein phosphatase (DUSP)] inactivates MAP-kinases and, thereby, inhibits activation of monocytes and macrophages in humans and mice. MKP-1 is up-regulated by pro-inflammatory stimuli and forms an important negative feedback loop to limit MAP-kinase signaling and pro-inflammatory mediator release (60, 101). MKP-1 is also up-regulated in thioglycolate-elicited peritoneal and bone marrow murine macrophages by GC treatment (44, 60). In MKP-1^−/−^ mice GC-mediated suppression of pro-inflammatory genes like IL-1β, TNFα, and COX-2 was markedly reduced and the inhibitory effect of GCs on zymosan-induced inflammation was abrogated (60). Mechanistically MKP-1 was shown to be involved in abrogation of the stabilizing effect of p38 MAPK on mRNAs coding for pro-inflammatory cytokines. In murine macrophages the regulation of the phosphorylation status of the mRNA destabilizing protein tristetraprolin seems to be a particularly important for this effect (102) (Figure 1, 13).

Bhattacharyy et al. (44) demonstrated a reduced survival rate in macrophage specific GR receptor knockout mice during LPS-induced septic shock. This effect was due to reduced up-regulation of MKP-1 by endogenous GCs and successive reduced MKP-1-mediated inactivation of p38 MAPK, since treatment of mice with a p38 MAPK inhibitor protected mice from LPS induced septic shock.

Thus, up-regulation of MKP-1 is another mechanism by which GCs inhibit pro-inflammatory mediator release by transactivation of gene transcription rather than by transrepression mechanisms.

## Other Anti-inflammatory Mediators Induced in Monocytes or Macrophages by GCs

The soluble decoy receptor for IL-1, IL-1R II inactivates IL-1 signals and has repeatedly been described to be up-regulated in human monocytes by GC treatment (59, 103). However, GCs also suppress the LPS-induced synthesis of the soluble IL-1 receptor antagonist in human monocytes (104). Thus, while GCs, at least in high concentrations, are usually effective in inflammatory disorders associated with increased IL-1 signaling like pustular psoriasis, Schnitzlers syndrome or adult onset Stills disease, the effects of GCs on IL-1 signaling *in vivo* have to be further evaluated. GCs have also been reported to induce synthesis of thymosin β 4 sulfoxide by human monocytes, which promotes wound healing and inhibits neutrophil migration *in vitro* and in carrageenan-induced inflammation *in vivo* (105).

Amelioration of acute lung injury by glucocorticoids in mice was shown to be mediated by induction of sphingosine kinase (SphK)1 in macrophages (106). The induction of SphK1 resulted in increased levels of sphingosine 1-phosphate, which enhanced barrier function of the lung endothelium by binding to sphingosine 1 receptor type 1 on endothelial cells (106).

ADAMTS2 (a disintegrin and metalloproteinase with thrombospondin motifs), a secreted metalloproteinase involved in wound repair, was reported to be specifically up-regulated in GC-treated human monocytes and may thus contribute to a GC-induced pro-resolution phenotype (107). Genome wide expression profiling identified other anti-inflammatory mediators which were up-regulated by GC treatment of human monocytes including adenosine receptor 3a and formyl peptide receptor (59). Similarly, a systematic analysis of mouse and human macrophages identified a robust induction of gene expression and a set of anti-inflammatory mediators (like Fkbp5, MKP-1, Tsc22d3, Per1) which were induced both in humans and mice (108). In summary, up-regulation of anti-inflammatory mediators in monocytic cells is an important global mechanism of GC action.

## GCs Effects on Phagocytosis

A hallmark of inflammatory resolution is the safe disposal of cellular debris and especially of apoptotic granulocytes. The uptake and subsequent removal of granulocytes by monocyte derived macrophages is called efferocytosis and is crucial for protecting tissues from exposure to inflammatory contents of dying cells (70, 109). Moreover, in contrast to other phagocytic processes, ingestion of apoptotic cells results in production of anti-inflammatory mediators like TGFβ or IL-10 and inhibition of pro-inflammatory mediator release, which further supports resolution of inflammation (110). It becomes increasingly evident that GCs are important regulators of this process. GCs increase the phagocytic capacity of human macrophages to engulf apoptotic cells (111, 112). This was associated with a loss of actin-containing podosome structures, reduced p130 Cas expression, loss of paxillin/pyk2 phosphorylation, and high levels of active Rac (112).

Interestingly endogenous GCs also seem to be involved in the augmentation of phagocytosis of apoptotic cells. Macrophages have been shown to express 11β-hydroxysteroid dehydrogenase, an enzyme responsible for conversion of inactive 11-dehydrocorticosterone in active GCs. Genetic depletion of the enzyme in mice was associated with delayed clearance of apoptotic neutrophils by different types of macrophages (113).

There is good evidence that the phagocytosis promoting capacity of GCs is due to GC-induced protein expression. Increased uptake of apoptotic granulocytes by GC-treated murine and human macrophages and human monocytes is mediated partly by GC-induced protein annexin A1 or milk-fat globule EGF factor 8 acting as opsonins binding phosphatidylserine or other molecules on apoptotic cells (71, 72, 114). Complement opsonization plays an important role in phagocytosis of apoptotic cells by the innate immune system. Interestingly, GCs have been demonstrated to induce production of phagocytosis promoting C1q by human monocytes and different rat macrophage populations (59, 115). A deficiency in uptake of apoptotic cells is seen in systemic lupus erythematosus (SLE) which has been linked to a reduced capacity of monocytes from SLE patients to produce C1q (116). This indicates a pathophysiological relevance of C1q produced by monocytes as well as generally C1q production in resolution of inflammation.

GC-regulated genes involved in promoting phagocytosis of apoptotic granulocytes include CD163 (84), MFGE8, and proto-oncogene tyrosine-protein kinase MER (MerTK) in human monocytes (59, 114). A role of MerTK in mediating GC-regulated induction of phagocytosis in human macrophages has been demonstrated using blocking antibodies and gene-silencing (117, 118). The scavenger receptor stabilin-1 has also been demonstrated to be increased upon GC treatment in human macrophages (119).

While GCs were shown to impair phagocytosis of adherent-invasive *Escherichia (E) coli* bacteria in a human monocytic cell-line (THP-1) (120) there are numerous reports demonstrating that in primary cells GCs not only increase the capacity for phagocytic uptake of apoptotic granulocytes but rather induce a general pro-phagocytic phenotype.

GC treated human monocytes and macrophages display an increased uptake of latex beads (59, 121) as well as an enhanced non-phlogistic uptake of myelin and *Staphylococcus (S) aureus* (122) and *E. coli* bacteria (shown in sheep monocytes) (121, 123).

GC-induced CD163 has been reported as an innate immune sensor for certain bacteria in human monocytes (*E. coli, S. aureus*) (124). Further studies demonstrated that soluble CD163 can directly bind to fibronectin bound to the staphylococcal surface and this enhances phagocytosis and also killing of bacteria while preventing human monocytes from overwhelming inflammatory response during staphylococcal infections (125, 126). GC-mediated induction of efferocytosis by lung macrophages in mice was also associated with increased uptake of *Streptococcus pneumoniae* but reduced bactericidal functions of these cells (127).

In conclusion, GC-treated monocytes are able to limit tissue damage due to a general higher capacity for efferocytosis and phagocytosis of pro-inflammatory stimuli, like microbial agents, particles and cellular debris, a process that has to be tightly controlled *in vivo* to ensure both effective antimicrobial responses and resolution of inflammation.

## GC Effects on Antigen Presentation and T-cell Activation

Monocytes and macrophages are able to actively participate in the induction of adaptive immune responses. They can present antigens to T cells and trigger polarized Th-cell responses (128–130) GC treatment of murine macrophages results in down-regulation of MHC class II expression (131). This phenomenon is also observed *in vivo*. Deactivation of circulating human monocytes is associated with loss of surface HLA-DR during sepsis and this correlates with high cortisol level (132). Moreover, expression of co-stimulatory molecules necessary for effective T cell stimulation—B7.1 and B7.2—is down-regulated by GCs which, in addition to altered cytokine production, contributes to diminished antigen presenting capacity of GC-treated human monocytes (133). Thus, GCs effectively reduce the capacity of monocytes to induce effector T-cell activation. In addition, GC-treated murine bone marrow monocytes also inhibited anti-CD3/CD28-induced proliferation of CD4^+^ and CD8^+^ T-cells *in vitro* and inhibited CD4^+^ T-cell-induced colitis in a therapeutic setting (134). The later was associated with a reduction of IFNγ and IL-17 secretion of effector T-cells restimulated *ex vivo* and induction of clusters of CD15^+^ Treg in colonic mucosa.

*In vitro* repetitive stimulation of naïve T-cells by GC-treated murine bone marrow monocytes resulted in generation of T-reg (134). GC-treated murine macrophages also stimulated T-reg differentiation *in vitro* which could be responsible for GC-mediated attenuation of acute lung injury (78) and together with GM-CSF in enhanced allo cardiac graft survival (135). This indicates that GC-treated monocytes have the dual ability to inhibit inflammation induced by effector T-cells and simultaneously induce tolerance by induction of T-reg. The interaction between GCs, monocytes/macrophages and T-cells may be even more complex, as in certain conditions (patients with rheumatoid arthritis) the presence of monocytes inhibits GC-mediated apoptosis of effector T-cells (136).

## GC Effects on Monocyte Adhesion and Migration

The GC treatment of human monocytes results in alterations of the cytoskeleton and decreased adherence to plastic surfaces (59, 112). Alterations of the cytoskeleton are not only important for adherence but also influence cell migration. An inhibitory effect of GCs on the migration of human monocytes in response to oxidized beta-very low density lipoprotein has been reported (137). *In vivo* GCs inhibited zymosan-induced extravasation of murine monocytes and macrophages which was partly dependent on annexin-1 expression (138) as well as MKP-1 expression (60). GCs also inhibited primary peritoneal macrophage migration into carrageenan-induced inflammation in mice (139).

However, GCs were also able to stimulate expression of chemokine receptors on human monocytes like the monocyte chemokine receptor CXCR4 (140). Moreover, GCs induced undirected cell migration (chemokinesis) as well as *in vitro* migration of human monocyte toward some chemotactic stimuli like fMLP (59) or C5a (141). Up-regulation of the fMLP receptor was shown to contribute to fMLP-induced chemotaxis of human monocytes (59). GCs were able to induce cell migration in murine ER-Hoxb8-immortalized bone marrow-derived macrophages lacking PPARγ, but not in control macrophages (53).

The different effects of GC treatment on monocyte and macrophage migration could be due to a differential activation of the analyzed cells. Thus, GCs may inhibit migration of pro-inflammatory activated monocytes while they may increase both differentiation of an anti-inflammatory monocyte subtype from naïve monocytes and their migration into inflamed tissue during resolution of inflammation (37). Experimental data from mice support this hypothesis. Indeed, GC-mediated attenuation of acute lung injury in mice was associated with reduced numbers of classical but increased numbers of alternatively activated macrophages in the lungs (78). Similarly, long-term treatment of mice with GCs was associated with increased infiltration of macrophages with an anti-inflammatory phenotype in adipose tissues (142).

## Action of GCs on Survival, Programmed Cell Death, and Differentiation of Anti-inflammatory Macrophages

Generally, at least in humans, monocytes are relatively short living cells, which die spontaneously in the absence of appropriate stimuli (143). Thus, their differentiation into macrophages and dendritic cells closely depends on the presence of appropriate survival signals. Pro-inflammatory factors like LPS or cytokines like IL-1β and TNFα are known to enhance the survival of pro-inflammatory monocytes (143) while much less is known about the molecular mechanisms promoting survival of anti-inflammatory monocytes.

There are conflicting results regarding the effects of GCs on monocyte/macrophage apoptosis. GCs have been reported to induce apoptosis in human monocytes after prolonged culture with low amounts of serum (144, 145). Suppression of IL-1β, which is required for monocyte survival and ligation of CD95 were reported to be involved in this effect. GCs also promoted apoptosis in LPS-stimulated rat alveolar macrophages (146).

Since no GC-induced apoptosis of human monocytes or macrophages was observed by others (59, 77, 112) the pro-apoptotic effects seem to be dependent on specific culture conditions and especially the activation status of the cells analyzed. GC treatment was able to antagonize biphosphonate-induced apoptosis in specialized murine macrophages (osteoclasts) (147). In pro-inflammatory human monocytes induced by GM-CSF, but not in M-CSF treated cells, GC treatment induced apoptosis via inhibition of ERK1/2 activity. Downregulation of ERK1/2 activity was associated with reduced activity of the p90 ribosomal-S6 kinase, reduced phosphorylation of Bcl-2-Antagonist of Cell Death (Bad) and increased caspase-3 activity which resulted in apoptosis (148) (Figure 1, 14). In a genome-wide expression screening of naïve human monocytes GCs were found to induce anti-apoptotic factors, like enzymes responsible for glutathione synthesis, while inhibiting expression of pro-apoptotic mediators (59). Consequently, GC-treated human monocytes were found to be protected from staurosporine-induced apoptosis which correlated with increased glutathione levels in GC-treated cells. The molecular mechanisms involved in protection from apoptosis in GC-treated human monocytes comprised activation of the ERK/MAPK kinase pathway induced by ligand binding to the adenosine A3 receptor (A3AR) following up-regulation of this receptor by GCs (149). Increased ERK1/2 signaling resulted in lower susceptibility to apoptosis accompanied by inhibition of caspase activity and induction of c-Myc–dependent anti-apoptotic genes (149) (Figure 1, 15).

*In vivo*, monocytopenia has been reported after GC treatment (150) in mice. However, more recent data indicate rather complex effects of GCs on monocytes in peripheral blood. In patients with uveitis, GC treatment was associated with increased numbers of CD14^++^CD16^+^ intermediate monocytes (151). However, in healthy donors, GCs increased levels of circulating human monocytes in general while depleting a subpopulation characterized by CD16 expression (152). Thus, the effect of GCs on the survival of different monocyte and macrophage subpopulations (e.g., CD16^+^ monocytes) *in vivo* warrants further detailed investigation. *In vitro*, it can be concluded that in humans GCs are able to protect naïve monocytes from apoptosis and thereby favor differentiation into an anti-inflammatory monocyte subtype while they induce apoptosis in pro-inflammatory activated monocytes via similar molecular pathways involving ERK1/2 (148, 149).

## The Action of GCs on Monocytes and Macrophages Depends on Differentiation as Well as Activation Status of These Cells

As described in detail above, GCs have both suppressive as well as enhancing effects on monocytes and macrophages. For an integrated view of GC action on these cells it is first of all important to keep effects on macrophages and monocytes apart. Moreover, in the recent years it has become evident that monocytes are no uniform precursor cells of macrophages and dendritic cells but display a substantial functional heterogeneity (153). Stimulation of monocytes with microbial TLR ligands results in pro-inflammatory mediator production, similar to macrophage activation, and this process is generally inhibited by GCs (Figure 2). However, there are marked differences regarding GC action on monocytes and macrophages. A recent systematic comparison of the effects of GCs on human monocytes vs. macrophages revealed that the transcriptome response differed in magnitude between both cell types. While 4 h GC treatment regulated 1,035 mRNAs in monocytes only 165 were regulated in macrophages (63). The majority of genes regulated in monocytes were involved in cell differentiation.

In agreement with these data, prolonged GC treatment of unstimulated human blood (59) or murine (154) bone marrow monocytes resulted in differentiation of cells with pro-resolution and anti-inflammatory phenotype characterized by expression of the scavenger receptor CD163 (Figure 2).

In the murine system, GC-induced bone marrow monocytes were systematically compared with the previously described peripheral blood monocyte subsets and represent a distinct subset characterized by a unique signature of cell-surface proteins (154). However, murine GC-induced monocytes show similarities to the so-called myeloid derived suppressor cells (MDSCs), indicating their involvement in not only innate but also adaptive immune functions (154). Indeed, murine GC-induced monocytes inhibited effector T-cell activation and induced regulatory T-cells *in vitro* and *in vivo* (134).

In human monocytes GC treatment induced expression of the monocyte subset marker CD16 *in vitro* (82, 151). Accordingly, GC treatment was associated with an increased proportion of CD14^++^CD16^+^ cells *in vivo*. An enhanced expression of CD163 (known to be robustly induced by GCs), however, was found both in CD16^+^ and CD16^−^ monocytes (151). Other cell surface markers and gene products were similarly expressed in CD14^++^CD16^+^ monocytes isolated from patients with and without GC therapy. Thus, the relation between GC-induced monocytes and peripheral blood monocyte subsets remains enigmatic. From a functional viewpoint, human GC-induced monocytes were protected from apoptosis *in vitro* and therefore this population could represent a long living functional phenotype *in vivo* which may be important for termination of inflammatory responses and induction of resolution of inflammation (37, 59) (Figure 2).

Concerning contrasting effects of GCs on macrophages, the activation and differentiation status of these cells is in our opinion the most important factor determining differential effects of GCs (Figure 3). Mostly, suppressive effects of GCs were studied in the context of classical activation, e.g., microbial stimulation of macrophages in the presence or absence of INFγ. However, it was long known that in macrophages not stimulated by pro-inflammatory mediators, GCs can also induce gene expression including anti-inflammatory proteins like IL-10 or annexin A1. With respect to the global transcriptional response it was recently shown that GC treatment was associated with an induction rather than repression of genes in both human and murine macrophages (108), indicating that GCs rather induce an active expression pattern than repress naïve macrophages. Indeed many “alternative” faces of macrophage activation have been discovered (11, 12, 30, 155) (Figure 3).

The treatment of “naïve” macrophages with IL-4 or IL-13 results in alternative macrophage activation, in contrast to classical activation achieved by IFNγ and LPS. Alternatively activated human macrophages up-regulate MHCII expression but inhibit rather than induce T-cell proliferation (156). IL-4 or IL-13 stimulated macrophages up-regulate mannose receptor, which stimulates endocytosis and antigen uptake and are involved in resolution of inflammation, wound healing and angiogenesis (11, 12, 155). It is now generally accepted that GC treatment also induces partly overlapping response patterns compared to alternative macrophage activation with IL-4 and IL-13 both in rodents and humans like increased expression of the mannose receptor (157) or CD163 (94). In addition, alternative pathways of macrophage activation have been also described for immune complexes and TLR agonists as well as adenosine A2 receptor ligands and TLR agonists.

However, these different phenotypes most likely represent functional plasticity rather than distinct differentiation subsets of macrophages (12). As outlined in the introduction the rigid system of macrophage polarization toward classical or alternative is being replaced by a multidimensional model of macrophage activation. Thus, instead of activated phenotypes it is preferable to understand the plasticity of macrophage responses to different stimuli as distinct macrophage activation programs (26, 27). Recently, the functional and transcriptional action of different alternative activation stimuli including GCs on human macrophage activation was systematically compared and the results confirm an even broader diversity of macrophage polarization than depicted in Figure 3 (30). In contrast to other alternatively activated macrophages, GC-generated human macrophages are very effective in phagocytic uptake of apoptotic cells. Thus, while GCs inhibit classical activation of macrophages, they show different effects on unstimulated macrophages and generate a specific anti-inflammatory macrophage subtype which seems to be important for the resolution of inflammation by uptake of apoptotic granulocytes and production of anti-inflammatory cytokines like TGFβ and IL-10 (12) (Figure 3). The physiological importance GC-induced alternative macrophage activation for resolution of inflammation and tissue repair is highlighted by the finding that mice which lack the GR in macrophages show increased mortality and deregulation of tissue repair in an experimental cardiac infarct model (158).

Even more physiological heterogeneity in the response of macrophages to GCs is possible, since many distinct tissue resident macrophages with different embryogenic origins have been characterized (21–25, 159). Up to now there is only very sparse information on GC effects on distinct tissue resident macrophages. Few data exist for alveolar macrophages: In mice, GC-mediated attenuation of acute lung injury was associated with increased pulmonal numbers of macrophages with an alternatively activated phenotype (78). In rats, GCs inhibited COX-2 expression and promoted apoptosis of LPS-activated alveolar macrophages (42, 146). In humans, GCs were able to suppress LPS-induced secretion of IL-6, TNFα, and CXCL8 in lung macrophages but no differences in GC susceptibility between healthy controls, smokers and patients with chronic obstructive pulmonary disease were found (160).

## Conclusion

As discussed in the last chapter, GCs can suppress or enhance functional properties of monocytes and macrophages. Moreover, there is clear evidence for distinct GC effects depending on the cell type (monocytes or macrophages and their subtypes in peripheral blood or tissues) or the activation status of these cells.

Therefore, regarding GC effects on monocytes and macrophages *in vivo*, it is obvious that models focused on specific molecular GC mechanisms like transrepression of pro-inflammatory mediators or induction of anti-inflammatory mediators are not sufficiently suited. However, despite the complexity of described GC effects on monocytes and macrophages an integrated view of the GC mode of action on monocytes and macrophages is possible when the physiological course of inflammatory responses *in vivo* is considered.

Thus, we propose the following model for differential GC action on monocytes and macrophages which is depicted in Figures 2, 3. During initiation of inflammation GCs inhibit activation of macrophages and also monocytes by microbial products or endogenous danger signals. This mechanism should be especially important within inflamed tissue.

However, in order to achieve resolution of inflammation more is needed than mere suppression of inflammation. Indeed, it has been demonstrated that resolution of inflammation does not occur passively but is rather an active and highly coordinated process (70, 109). Simultaneosuly to inhibition of monocyte and macrophage pro-inflammatory activation GCs could act on “naïve” monocytes and induce differentiation of a long-lived pro-resolution phenotype. These GC-induced monocytes can migrate toward inflamed tissues and fulfill important functions in resolution of inflammation. GC-induced monocytes could differentiate into macrophages after tissue entry or could represent a unique stable differentiation status. While the fate of GC-treated monocytes in inflamed tissue is currently unknown, it is obvious that they share functionally similarities with GC-induced macrophages. Like these macrophages, GC-treated monocytes show increased phagocytic capacity for apoptotic cells and secrete anti-inflammatory and pro-resolution mediators. Thus, endogenous GCs, either released systemically during inflammation or generated locally from inactive precursors by action of 17-β-hydroxysteroid dehydrogenase (113), represent an important mechanism by which the amplification phase of inflammation is shifted toward resolution of inflammation. This contributes to protection from overwhelming inflammation and prevents establishment of chronic inflammatory processes.

Similarly, GC therapy could have a dual mode of action with inhibition of pro-inflammatory macrophage and monocyte activation and simultaneously inducing resolution of inflammation via action on naïve monocytes and macrophages. The latter would involve generation of long-living GC-induced monocytes which may be relevant for prolonged anti-inflammatory effects of GC treatment *in vivo*.

Further investigation of these mechanisms could lead to the development of novel therapeutic strategies to modulate undesirable inflammation in autoimmune disorders, including those of the skin, with fewer side effects via induction of inflammatory resolution rather than immunosuppression.

## Author Contributions

All authors listed have made a substantial, direct and intellectual contribution to the work, and approved it for publication.

### Conflict of Interest Statement

The authors declare that the research was conducted in the absence of any commercial or financial relationships that could be construed as a potential conflict of interest.
